# Dilated episcleral veins with secondary open angle glaucoma

**DOI:** 10.4103/0301-4738.77045

**Published:** 2011

**Authors:** Rajul S Parikh, Savari Desai, Kulin Kothari

**Affiliations:** Department of Glaucoma, Bombay City Eye Institute & Research Centre, Mumbai, India

**Keywords:** Choroidal effusion, dilated episcleral vein, partial thickness sclerectomy with sclerotomy, secondary open angle glaucoma, trabeculectomy

## Abstract

We report a case of dilated episcleral vein with secondary open angle glaucoma. A 65-year-old female presented with redness of both the eyes without any prior systemic history. Her ocular examination revealed dilated episcleral veins and a high intraocular pressure (IOP) of 38 mm Hg in the right eye. Systemic examination was negative for carotid cavernous fistula, low-grade dural arteriovenous fistula, dysthyroid ophthalmopathy, and primary pulmonary hypertension. During follow-up, her IOP remained in high thirties despite maximum medications. She underwent right eye trabeculectomy with partial thickness sclerectomy with sclerotomy. In the beginning, the sclerotomy incision was not deepened into the suprachoroidal space. She developed choroidal effusion during surgery and the sclerotomy was deepened into suprachoroidal space and straw color fluid was drained. Postoperatively, she developed choroidal effusion, which resolved with conservative management. This case highlights the importance of sclerotomy in such cases of high episcleral venous pressure.

There can be various causes for increase in episcleral venous pressure, including venous obstruction, arteriovenous shunts or fistulas, Sturge-Weber syndrome, scleritis, dysthyroid orbitopathy, orbital tumors and idiopathic dilated episcleral veins (IDEV).[1–8] Secondary glaucoma related to dilated episcleral veins is difficult to manage medically and surgical complications are also high.[9] We report a 65-year-old woman with dilated episcleral vein of unknown etiology with secondary open angle glaucoma of only 1 year duration.

## Case Report

A 65-year-old female visited our institute in January 2007 with the chief complaint of redness of both the eyes. She had noticed redness of eye for last 9-10 months. She was a known case of hypertension for last 12 years.

**Figure 1:**
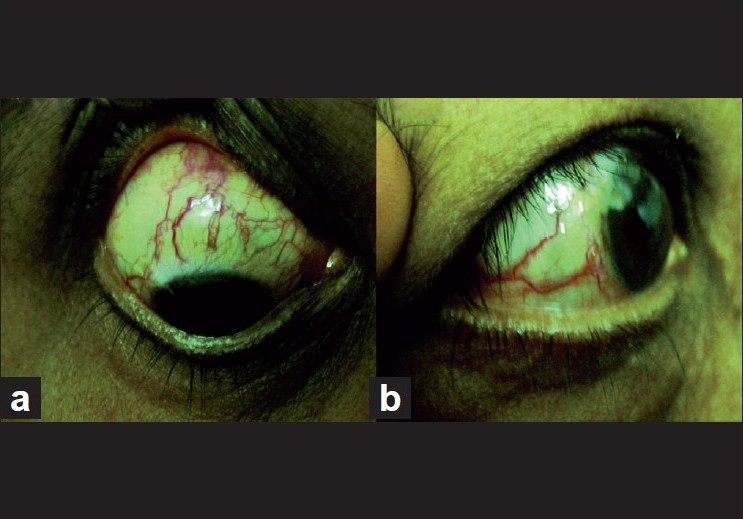
(a, b) Anterior and lateral views of the RE, showing dilated episcleral veins

On ocular examination, her best-corrected visual acuity was 20/20, N6 in both the eyes. Intraocular pressure (IOP) by applanation tonometry was 38 mm Hg in the right eye (RE) and 24 mm Hg in the left eye (LE). Anterior segment examination showed dilated episcleral vessels in both the eyes [Fig. 1a and b]. The RE episcleral veins were more dilated compared to that of LE. Gonioscopy showed open angles with blood in Schlemm’s canal in both the eyes. Dilated fundus examination showed cup to disc ratio (CDR) to be 0.6 with inferior rim thinning in the RE. In the LE, CDR was 0.5 with healthy neuroretinal rim. Peripheral retina showed normal caliber retinal vessels with no sign of choroidal hemangioma. Central corneal thickness was 588 μm in the RE and 586 μm in the LE. Automated perimerty (HFA 24-2) showed normal field. As her IOP was high, she was diagnosed as having ocular hypertension and was started on timolol 0.5% for both the eyes (BE) and Latanoprost eye drop at night in the RE.

She was examined again after 3 days by an orbit specialist. Ocular examination was negative for any signs suggestive of thyroid eye disease. The ultrasound (USG) of orbit showed normal caliber of superior ophthalmic vein in both the eyes. Her thyroid profile (T3, T4, TSH, TSH receptor antibody) was repeated twice and was normal. Her magnetic resonance imaging (MRI) and magnetic resonance angiography (MRA) orbit was not suggestive of carotid cavernous fistula (CCF) and low flow dural arteriovenous fistula (AV fistula). Provisional diagnosis of idiopathic elevated episcleral venous pressure was made. She was also given a course of systemic steroid (Tab Omnacortil 60 mg once a day with tapering of 10 mg dosage every week) in tapering dose, which did not reduce dilated episcleral vein.

During follow-up visit, her IOP always remained in high thirties in the RE and mid-twenties in the LE. She was given in addition topical alpa-2 agonist (brimonidine tartarate three times a day) and topical carbonic anhydrase inhibitor (CAI) (dorzolamide twice a day) in the RE. On maximum medical therapy, her IOP fluctuated between 32 and 42 mm Hg in the RE and 24 and 32 mm Hg in the LE between Jan 2007 and May 2007. In May 2007, her repeat visual field, optic disk and retinal nerve fiber layer (RNFL) scanning with Optical Coherence Tomography (OCT) findings were similar to her first visit findings. In view of her persistent elevated IOP and dilated episcleral veins, she underwent digital subtraction angiography (DSA) to rule out low-grade dural AV fistula. The DSA showed normal brain vasculature. She was asked to have the opinion of a chest physician to rule out primary pulmonary hypertension and/or tumor at the apex of lung. The chest X-ray and computerized tomography (CT scan) of chest were normal.

In December 2007, as her IOP was always in high thirties in RE despite maximum medications, she was advised trabeculectomy. It was thought that due to high episcleral venous pressure, she would have high chances of uveal effusion after trabeculectomy, so partial thickness sclerectomy with sclerotomy in infero-temporal region was planned. Right eye trabeculectomy with partial thickness sclerectomy with sclerotomy in infero-temporal region was performed on 7 January 2008. Initially, partial thickness sclerectomy with sclerotomy was performed; however, the incision was not deepened into the suprachoroidal space. Subsequently, routine fornix based trabeculetomy with mitomycin C (large surface area technique) was performed superiorly. After completion of trabeculectomy, there was difficulty in forming the anterior chamber. It was thought that the patient has developed chroidal effusion and the sclerotomy was deepened into suprachoroidal space and straw color fluid was drained. After the choroidal fluid drainage, the anterior chamber was formed.

Postoperatively, on day 1, her IOP was 12 mm Hg, and she had a good bleb and shallow anterior chamber with collerate iridocorneal touch. On fundus examination, she had bullous choridal detachment which was documented on ultrasonography. As the choroidals were not kissing and central anterior chamber was formed, she was treated conservatively. She was given systemic steroids for 15 days. After 3 weeks of surgery, her choroidals subsided with conservative management. Her IOPs on 23rd day of surgery were 20 and 25 mm Hg in the RE and LE, respectively. On anterior segment examination, she had a good bleb and well-formed anterior chamber. Seven months after the surgery, when she was seen last, her IOP was 22 mm Hg in the RE without any medicines and 25 mm Hg in the LE on anti-glaucoma medications.

## Discussion

Dilated episcleral vein with secondary glaucoma is always difficult to diagnose and manage. IDEV is considered congenital in origin and diagnosis of exclusion. However, all pathologies which lead to dilated episcleral vein require non-invasive or invasive investigations for diagnosis. In patients with IDEV, a congenital abnormality in vasculature and familial predisposition have been suggested as the cause;[6] however, some reports suggest that the ocular injection is acquired.[7] Most of the studies in literature report this entity in the second or third decade. It is actually a diagnosis of exclusion; however, in our case, the presentation was late and despite normal investigation we do not think it could be IDEV. Radius-Maumenee syndrome is another possibility but it is usually unilateral and the episcleral veins usually regress in caliber and congestion after trabeculectomy, which did not happen in our case. Low AV grade fistula would come very close to as a first differential. The gold standard to rule out the presence of a low-grade dural AV fistula is an angiography as in a small percentage of cases; it can be missed on MRA. Both MRA and DSA were normal and we could not confirm the diagnosis. However, it is possible that a low-grade fistula can spontaneously get closed and does not show any abnormality on angiography. We did not measure the episcleral blood flow or episcleral venous pressure in our patient, which we feel, would not have led to any specific diagnosis.

The management is always difficult in these cases. Some argue for early filtration surgery. It is argued that drug that works on uveoscleral pathway may be able to reduce better than a aqueous suppressant. However, in our patient, prostaglandin analogue did not reduce IOP. It is thought that due to high episcleral venous pressure, the surgery would have higher complication rate. We assumed that higher episcleral venous pressure and sudden IOP lowering after trabeculectomy would lead to a uveal effusion like syndrome.[9] From this perspective, we decided partial thickness sclerectomy and sclerotomy during trabeculectomy to take care of cilliochoroidal detachment and choroidal effusion. However, we thought that entering into the suprachoroidal space before trabeculectomy will lead to hypotony and will make surgery more difficult and did not enter into suprachoroidal space. This led to sudden hypotony and development of choroidal effusion intraoperatively which resolved very slowly.

To conclude, this case highlights that in some cases with dilated episcleral veins, we may not be able to diagnose the exact etiology despite thorough investigation. It also highlights the importance of sclerotomy in such cases of high episcleral venous pressure. It may be better to do sclerotomy and enter the suprachoroidal space before trabeculectomy as sudden hypotony after trabeculectomy may lead to transudation of fluid into suprachoroidal space and massive choroidal effusion during surgery.
